# Triple Negative Breast Cancer: Molecular Subtype-Specific Immune Landscapes with Therapeutic Implications

**DOI:** 10.3390/cancers16112094

**Published:** 2024-05-31

**Authors:** Antonia Syrnioti, Stamatios Petousis, Lisa A. Newman, Chrysoula Margioula-Siarkou, Theodora Papamitsou, Konstantinos Dinas, Triantafyllia Koletsa

**Affiliations:** 1 Department of Pathology, School of Medicine, Aristotle University of Thessaloniki, 54124 Thessaloniki, Greece; asyrnioti@auth.gr; 2 2nd Department of Obstetrics and Gynaecology, Aristotle University of Thessaloniki, 54124 Thessaloniki, Greece; petustam@auth.gr (S.P.); dinas@auth.gr (K.D.); 3 Department of Breast Surgery, New York Presbyterian-Weill Cornell Medicine, New York, NY 10065, USA; lan4002@med.cornell.edu; 4 MSc Program in Gynaecologic Oncology and Breast Oncology, Aristotle University of Thessaloniki, 54124 Thessaloniki, Greece; margioulasiarkouc@auth.gr; 5 Laboratory of Histology-Embryology, School of Medicine, Aristotle University of Thessaloniki, 54124 Thessaloniki, Greece; thpapami@auth.gr

**Keywords:** triple negative breast cancer, TNBC, tumor-suppressive microenvironment, tumor-promoting microenvironment, immune checkpoint inhibitors, targeted therapy

## Abstract

**Simple Summary:**

Triple Negative Breast Cancer (TNBC) comprises approximately 15–20% of all breast cancer (BC) cases, is often diagnosed at an advanced stage, and is generally associated with an adverse clinical outcome. This systematic review explores differences in the tumor immune microenvironment (TIME) across various molecular subtypes of TNBC. Six studies meeting our inclusion criteria were analyzed, revealing diverse TIMEs with distinct compositions among TNBC molecular subtypes. The IM subtype shows robust immune infiltration, while LAR and MSL subtypes display more immunosuppressive milieu. The spatial distribution of immune cells and immune checkpoint expression varies across TNBC molecular subtypes. TIME heterogeneity reflects genomic diversity, along with differential signaling pathways and metabolic activation. Understanding TIME variability offers strategic opportunities for personalized therapeutic interventions in TNBC.

**Abstract:**

Triple Negative Breast Cancer (TNBC) is characterized by distinct molecular subtypes with unique biological and clinical features. This systematic review aimed to identify articles examining the differences in the tumor immune microenvironment (TIME) across different TNBC molecular subtypes. Six studies meeting inclusion criteria were analyzed, utilizing gene expression profiling and bioinformatic analyses to classify TNBC samples into molecular subtypes, as well as immunohistochemistry and cell deconvolution methods to characterize the TIME. Results revealed significant heterogeneity in immune cell composition among TNBC subtypes, with the immunomodulatory (IM) subtype demonstrating robust immune infiltration, composed mainly of adaptive immune cells along with an increased density of CTLA-4+ and PD-1+ TILs, high PD-L1 tumor cell expression, and upregulation of FOXP3+ Tregs. A more immunosuppressive TIME with a predominance of innate immune cells and lower levels of tumor-infiltrating lymphocytes (TILs) was observed in luminal androgen receptor (LAR) tumors. In mesenchymal stem-like (MSL) tumors, the TIME was mainly composed of innate immune cells, with a high number of M2 tumor-associated macrophages (TAMs), while the BL and M tumors displayed poor adaptive and innate immune responses, indicating an “immune-cold” phenotype. Differential activation of signaling pathways, genomic diversity, and metabolic reprogramming were identified as contributors to TIME heterogeneity. Understanding this interplay is crucial for tailoring therapeutic strategies, especially regarding immunotherapy.

## 1. Introduction

Triple Negative Breast Cancer (TNBC) constitutes around 15–20% of all breast cancer (BC) cases, typically presents at an advanced stage, and is associated with a relatively poor prognosis [1,2,3]. TNBC is considered a diverse group of neoplasms comprising multiple molecular subtypes. Specifically, in 2011, Lehmann et al. proposed six molecular subtypes of TNBC: two basal-like subtypes (referred to as BL1 and BL2), along with an immunomodulatory (IM), a mesenchymal (M), a mesenchymal stem-like (MSL), and a luminal androgen receptor (LAR) subtype [4]. Following Lehmann’s classification, numerous studies have used gene expression profiling to document concordant molecular classifications of TNBC. Burstein et al. proposed four molecular TNBC subtypes, including a basal-like immune-activated (BLIA), a basal-like immunosuppressed (BLIS), a mesenchymal (MES), and a LAR subtype [5]. Liu et al. suggested the Fudan University Shanghai Cancer Center (FUSCC) classification system, which comprised four molecular TNBC subtypes, including LAR, IM, a mesenchymal-like (MES), and a basal-like and immune-suppressed (BLIS) subtype [6].

Notably, each molecular subtype of TNBC exhibits distinct biological characteristics [7,8]. The BL1 subtype is marked by the upregulation of genes related to the cell cycle and DNA damage response, while the BL2 subtype exhibits enrichment in growth factor signaling, glycolysis, and gluconeogenesis [9]. A hallmark of both the M and MSL subtypes is the high expression of genes related to motility and epithelial-mesenchymal transition (EMT). The MSL subtype additionally exhibits diminished expression of proliferation-related genes, while also being enriched with genes associated with mesenchymal stem cells [9]. The IM subtype is characterized by the abundant expression of genes associated with immune cell processes, including antigen presentation and immune signal transduction pathways [9]. The LAR subtype preferentially expresses genes involved in steroid synthesis, porphyrin metabolism, androgen/estrogen metabolism, and Peroxisome proliferator-activated receptor (PPAR) signaling [6,10]. This diversity translates into a broad spectrum of clinical outcomes, and responses to treatment. Importantly, evidence suggests that TNBC molecular subtypes exhibit variations in their response to standard neoadjuvant chemotherapy, with the BL1 subtype showing the highest probability of achieving a pathological complete response (pCR) [11]. In addition, both M and MSL subtypes have exhibited responsiveness to Phosphoinositide 3-kinase (PI3K)/mammalian target of rapamycin (mTOR) inhibitors and abl/src inhibitors [4], whereas LAR tumors might benefit from androgen receptor (AR) blockade [12]. This highlights the challenge of establishing consistently effective treatment regimens [13]. Moreover, the reproducibility of their identification across different transcriptional methodologies underscores the potential value of incorporating these molecular subtypes into prognostic stratification and personalized medicine for TNBC [4,5,6].

At the same time, emerging insights shed light on the distinct nature of the tumor immune microenvironment (TIME) in TNBC, which is overall characterized by a higher number of immune cells, particularly tumor-infiltrating lymphocytes (TILs), compared to other invasive BC subtypes [14,15,16]. Additionally, research indicates that the TIME significantly impacts the development, expansion, and spread of TNBC [17,18,19,20]. However, little is known regarding the heterogeneity of the TIME between different molecular subtypes of TNBC. Therefore, gaining a deeper understanding of the TNBC TIME is imperative for accurately predicting outcomes and devising effective treatments. In this systematic review, we aimed to identify original articles investigating the differences in the composition of the TIME between various molecular subtypes of TNBC.

## 2. Material and Methods

### 2.1. Search Strategy

This systematic review was conducted according to the Preferred Reporting Items for Systematic Reviews and Meta-Analyses (PRISMA) guidelines [21] based on a protocol registered in the OSF database (registration number: https://osf.io/h9f6m/, accessed on 8 May 2024). A search of the PubMed, Scopus, ClinicalTrials.gov, Cochrane library, and Trip databases was performed from 2011, when TNBC molecular subtyping was first published [4], to December 2023. The Medical Subject Headings (MeSH) and search terms that were used are the following: “TNBC” AND “Molecular subtypes” AND “Microenvironment”.

### 2.2. Study Selection and Data Collection Process

Two authors (AS, TK) independently performed the search, reviewed the search results, and assessed them for eligibility according to predetermined inclusion and exclusion criteria. Specifically, eligible studies were considered to be those (i) providing information for both the molecular subtypes of TNBC and immune microenvironment, (ii) using one of Lehmann’s, Burstein’s, or Liu’s TNBC molecular subtypes, (iii) written in the English language, and (iv) published during 2011–2023. On the other hand, the studies that met one of the following criteria were excluded: (i) narrative reviews, (ii) case reports.

**Figure 1:**
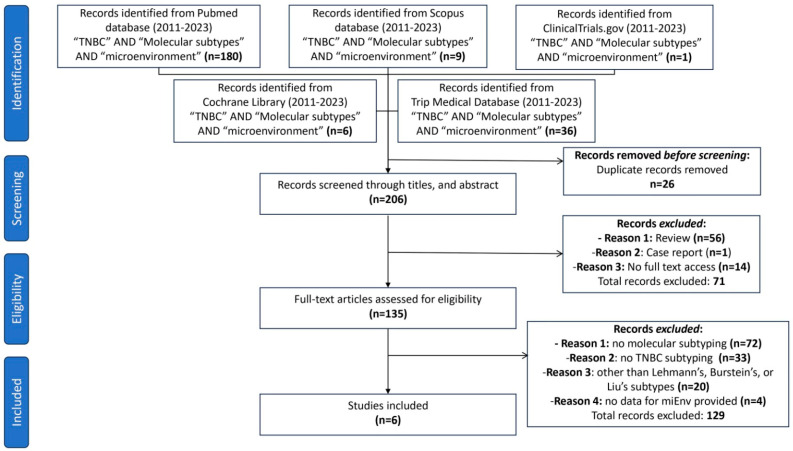
Flow diagram of the study selection process.

Each author independently reviewed abstracts and compiled a list of studies for full-text review. A comparison was made between the lists, and any differences were resolved through consensus. The screening process of the articles is illustrated in detail in Figure 1.

### 2.3. Primary and Secondary Outcomes

Primary outcomes of this study were the associations of TNBC molecular subtypes with specific TIME profiles. Secondary outcomes included differences in the spatial distribution of immune cells in the tumor microenvironment, as well as immune checkpoint (IC) expression across various molecular subtypes of TNBC.

## 3. Results

In total, 232 publications were retrieved from the PubMed, Scopus, ClinicalTrials.gov, Cochrane Library, and Trip databases. Twenty-six duplicate articles were removed. After screening the remaining 206 titles and the abstracts, 56 reviews, 1 case report, and 14 articles with no access to the full text were excluded. The remaining 135 articles were retrieved and underwent full-text screening. Subsequently, 72 studies were ruled out as they included no molecular subtyping at all, 33 studies harbored no TNBC subtyping, and 20 studies used alternative TNBC classifications (other than those proposed by Lehmann, Burstein, or Liu et al.). Of the remaining 10 studies, 4 provided no information for the TIME. Finally, six studies were selected for this systematic review. These studies were published in a period from 2020 to 2022 (Table 1).

### 3.1. Methodology Used for TNBC and TIME Subtyping

The six selected studies included a total of 3758 TNBC samples. Two of the six studies [25,27] corresponded to original molecular investigation, whereas the other four [22,23,24,26] utilized data from databases. In either case, gene expression profiling analysis had been conducted utilizing microarray, RNA-sequencing, or whole-exome sequencing data. Bioinformatic analysis was subsequently performed to classify the cases into molecular subtypes.

Specifically, Bareche et al. [22] categorized a portion of the samples according to Lehmann’s molecular classification, encompassing BL1 and BL2, IM, LAR, M, and MSL subtypes, another subset of the samples based on Jiang’s molecular classification [28], which corresponded to the FUSCC classification system, including BLIS, IM, LAR, and MES subtypes, and a third portion of the samples according to Burstein’s molecular classification, encompassing BLIS, BLIA, LAR, and MES subtypes. In Kim et al.’ study [23], the molecular subtypes assigned encompassed stem-like (SL), MSL, LAR, and IM TNBCs. Both Rodríguez-Bautista and Zhang et al. [24,25] contrasted the IM subtype with non-IM TNBC, including BL1, BL2, M, MSL, LAR, and unspecified (UNS) subtypes. Thompson et al. [26] developed a LAR-gene signature and contrasted the LAR from non-LAR TNBC subtypes. Finally, Suntiparpluacha et al. [27] categorized the samples into four subgroups, with three of them aligning with the LAR, BL2, and M subtypes as defined by Lehmann’s TNBC classification.

For TIME analysis, five of the six studies [22,23,24,26,27] employed various cell deconvolution methodologies, such as CIBERSORT and Tumor IMmune Estimation Resource (TIMER) Database Analysis. These approaches leverage established gene expression signatures linked to distinct cell types, enabling the characterization and quantification of the relative abundance of these cells within the sampled tissues. In contrast, Rodríguez-Bautista et al. [25] utilized immunohistochemistry (IHC) to delineate the composition of immune cells within the microenvironment. Overall, the immune cells investigated included B-cells, Natural killer (NK) cells, T-cells [Central memory (Tcm), Effector memory (Tem), Follicular helper (Tfh), CD4+ Helper (Th1 and Th2), CD8+, gamma delta (γδ), and regulatory T-cells (Tregs)], plasma cells, activated and inactivated dendritic cells (aDCs and iDCs, respectively), neutrophils, eosinophils, mast cells, M1 and M2 tumor-associated macrophages (TAMs), and myeloid-derived suppressor cells (MDSCs).

In addition, four studies [23,24,25,27] examined differences in IC expression across various molecular subtypes of TNBC. Among these, Rodríguez-Bautista et al. [25] utilized IHC for PD-L1, and CTLA-4, while Kim et al. [23], Zhang et al. [24], and Suntiparpluacha et al. [27] relied on gene expression analysis data for PD-L1, PD-1, and/or CTLA-4 immune checkpoints.

### 3.2. Associations of TNBC Molecular Subtypes with Specific TIME Profiles

Three out of the six studies [22,23,25] concluded that the IM subtype exhibited an immune-rich microenvironment characterized mainly by an increased number of adaptive immune cells, such as CD8+, CD4+ T-cells, and B-cells, as well as γδ T-cells, NK cells [22,23,25], and FOXP3+ cells [25]. In addition, the “immune-hot” status in the IM subtype was affirmed by the upregulation of eight immune-related hub genes, including *BIRC3*, *BTN3A1*, *CSF2RB*, *GIMAP7*, *GZMB*, *HCLS1*, *LCP2*, and *SELL* [24].

On the other hand, LAR tumors exhibited a more immunosuppressive microenvironment represented predominantly by innate immune cells, such as mast cells, and iDCs [22], lower levels of TILs with comparatively increased numbers of CD4+ and CD8+ T-cells, and reduced Tregs and cycling T-cells [26]. Activation of MDSCs, as well as diminished priming and activation of immune cells and IFN-γ signaling, were also observed among LAR samples [26].

The immune contexture in the MSL subtype presented mainly cells of innate immunity, including neutrophils, eosinophils, mast cells, iDCs, and NK cells [22], along with a high number of M2 TAMs [23].

Both BL and M subtypes demonstrated poor adaptive and innate immune responses, suggesting an “immune-cold” phenotype [22]. In contrast, Suntiparpluacha et al. mentioned high CD8+ T-cells in BL2 and M subtypes [27]. It is emphasized that they utilized an indirect methodology for TNBC categorization in Lehmann’s subtypes, possibly explaining the inconsistent results. However, they also implied an immunosuppressive microenvironment of LAR, M, and BL2 TNBCs.

### 3.3. Spatial Distribution of Immune Cells and IC Expression

As regards the spatial distribution of immune cells in the tumor microenvironment, there were differences between TNBC subtypes. The IM subtype displayed a fully inflamed (FI) pattern, indicative of “immune-hot” TIME, whereas immune cells in the MSL and LAR subtypes exhibited a margin-restricted (MR) spatial distribution pattern. A predominantly stroma-restricted (SR) pattern was observed in the BL subtype [22]. Finally, regarding the IC expression, the IM subtype exhibited an increased density of CTLA-4+ and PD-1+ TILs and an enrichment of PD-L1, PD-1, and CTLA-4 genes, along with high PD-L1 expression by tumor cells [22,23]. In contrast, Suntiparpluacha et al. described an elevated expression of PD-1 and PD-L1 in the BL2 subgroup [27].

### 3.4. Differences in Signaling Pathways, Metabolic Activity and Genomic and Transcriptomic Diversity

Interestingly, IM and LAR tumors were observed to exhibit an upregulation in the interferon-alpha (IFNα), interferon-gamma (IFNγ), IL6–JAK–STAT3, and IL2–STAT5 signaling pathways [23], while an enrichment of the Wnt/β-catenin pathway was reported among IM tumors [25]. Several long non-coding RNAs (lncRNAs), including LINC00173, LINC00854, LINC00869, LINC00426, LINC00861, LINC01550, and LINC00312, were also differentially expressed in IM vs. non-IM tumors [25]. Furthermore, BL and M tumors were found to exhibit high chromosomal instability (CIN) and copy number loss in the 5q and 15q chromosomal regions, encompassing major histocompatibility complex (MHC)-related genes [22]. Concurrently, Kim et al., and Bareche et al., highlighted an upregulation in glycolysis, and lipid metabolism, respectively, among LAR tumors [22,23]. Nevertheless, it is worth noting that in the study conducted by Thompson et al., LAR tumors displayed a downregulation of glycolysis [26].

## 4. Discussion

The systematic review presented herein sheds light on the intricate relationship between TNBC and its TIME, focusing on the variability across different molecular subtypes. Overall, our results show significant heterogeneity in the immune cell composition characterizing each TNBC molecular subtype. Specifically, the IM subtype exhibited a robust immune infiltration, along with a FI spatial distribution pattern, an increased density of CTLA-4+ and PD-1+ TILs, and high PD-L1 expression by tumor cells, indicative of “immune-hot” tumors. MSL and LAR subtypes showed a more immunosuppressive milieu, whereas BL and M could be considered as “immune cold” tumors demonstrating a protumorigenic TIME with poor adaptive and innate immune responses, along with a predominantly SR and MR pattern, respectively.

Of note, IM showed elevated levels of TILs including increased FOXP3+ Tregs compared to non-IM tumors. Traditionally, Tregs have been associated with unfavorable clinical outcomes in invasive BC, potentially due to the immunosuppressive microenvironment fostered by transforming growth factor (TGF-β) and interleukin (IL)-2 stimuli [29,30]. However, more recent research has shown contradictory results in various types of tumors, including TNBC [31,32], possibly due to their abundance in robust immune responses. In this context, Tregs may act as a favorable prognosticator in the IM subtype, particularly when accompanied by increased CD8+ T-cells and CD20+ B-cells [32].

Various factors could potentially contribute to the TIME heterogeneity between different TNBC molecular subtypes, including differential activation of signaling pathways, genomic and transcriptomic diversity, as well as differences in metabolic reprogramming. For example, the upregulation of interferon-alpha (IFNα), interferon-gamma (IFNγ), IL6–JAK–STAT3, and IL2–STAT5 signaling pathways in IM and LAR tumors could contribute to enhanced immune cell infiltration [23]. Interestingly, though, the Wnt/β-catenin pathway, which was also enriched among IM tumors [11], has been found to interfere with tumor T-cell infiltration [33,34]. Additionally, some of the lncRNAs associated with IM tumors [25] have been implicated in immune response modulation, either by facilitating CD8+ TILs’ infiltration or by regulating the expression of PD-1 and CTLA-4 [35,36,37]. Furthermore, the high CIN, along with the copy number loss in the 5q and 15q chromosomal regions encompassing MHC-related genes, in BL and M tumors [22] may contribute to diminished cytotoxic activity, as well as reduced tumor antigenicity, potentially facilitating immune evasion [38]. Differences in metabolic activity may also play a role in the diversity of the TIME across various molecular subtypes. Specifically, the metabolic reprogramming observed in LAR tumors, with the upregulation in glycolysis and lipid metabolism [22,23], aligns with recent research showing that the accumulation of lactic acid and other lipid-derived metabolites inhibits the function of various pro-inflammatory immune cells, such as cytotoxic T-cells, while also promoting the expansion of immunosuppressive cell populations like MDSCs and M2 TAMs [39,40]. Nonetheless, the contrasting findings in the study by Thompson et al., which reported a downregulation of glycolysis in LAR tumors, emphasize the complexity of metabolic reprogramming in TNBC and suggest that further investigation is necessary to fully understand these dynamics [26].

Furthermore, it is crucial to highlight recent evidence indicating that TNBC TIME may vary among different populations, including those with African ancestry, where TNBC is more prevalent and associated with a higher mortality rate [41,42]. In a study by Martini et al., where RNA sequencing was conducted on a cohort comprising African Americans (AA) and West and East Africans with TNBC, a significantly higher number of intratumoral CD8+ memory T cells and Tregs, along with significantly elevated levels of stromal plasma cells, CD4+ and CD8+ memory T cells, and Tregs, was noted among AA individuals. Importantly, racial differences in gene expression were linked with distinct immune response signatures, implicating TIME variability as a potential contributor to clinical outcome disparities [41].

In recent years, it has been evident that the literature demonstrates a growing interest in the interactions between neoplastic cells and the microenvironment, with a recognition of tumors as complex ecosystems. Tumor immune contexture proved to be of clinical relevance [43,44], and the terms “hot” and “cold” tumors are widely used [22,44] with potential therapeutic implications. Cancer treatment can be optimized by taking into account both the characteristics of the neoplastic cells and TIME. Along this line of reasoning, the IM and BLIA TNBC subtypes have an “immune-hot” microenvironment and demonstrate high expression of immune-related genes, suggesting a potential for favorable response to IC blockade therapy, including agents targeting PD-1, PD-L1, and CTLA-4 [22,44,45,46,47]. Several clinical trials have investigated responses to IC blockade therapy in relation to various TNBC subtypes in a retrospective manner. The BLIA and LAR subtypes have been shown to benefit the most from atezolizumab in the phase I PCD4989 g trial, which included a metastatic TNBC (mTNBC) cohort treated with atezolizumab monotherapy. These subtypes were distinguished from M and BLIS tumors by higher levels of immune biomarkers (TILs, PD-L1, and IHC CD8 expression) [48]. Notably, Xiao et al. conducted a thorough multi-omics analysis of TNBC, with a primary focus on the delineation of three distinct TIME clusters, in an effort to personalize immunotherapy for TNBC patients, and then they identified the predominant molecular subtype in each TIME cluster. Specifically, they proposed TNBC classification into an “immune-desert” cluster, primarily constituted by BLIS subtype tumors with limited immune cell infiltration, an “innate immune-inactivated” cluster, predominantly composed of tumors with MSL features showcasing quiescent innate immune cells, and an “immune-inflamed” cluster, primarily featuring IM subtype tumors marked by prominent adaptive and innate immune cell infiltration, along with high IC expression. Their findings also suggested that individuals within the ‘immune-inflamed’ cluster may derive greater benefit from IC blockade therapies [49]. In addition, FUSCC has recently launched the Fudan University Shanghai Cancer Center TNBC Umbrella (FUTURE) trial for precision treatment of refractory mTNBC. In this prospective phase II umbrella clinical trial, based on the combination of FUSCC genomic sequencing and surrogate IHC-based subtyping, sustained effectiveness of anti-PD-1 blockade treatment along with nab-paclitaxel was observed among patients with IM tumors, taking into consideration PD-L1 and CD8 positivity (CD8-positive T-cells > 20%) [50,51]. This was further demonstrated in the phase II FUTURE-C-PLUS clinical trial [52], while a subsequent randomized controlled phase III FUTURE-SUPER trial (NCT05134194) is ongoing to validate these findings. In another trial, LAR and M tumors were linked to a considerably reduced pCR rate compared to BL1 tumors, regardless of the amounts of stromal TILs and PD-L1 expression [53]. This aligns with previous findings indicating that PD-L1 status is not entirely reliable as a biomarker for predicting response to immunotherapy [54,55]. Moreover, the impact of heterogeneity in the spatial distribution of immune cells in the TIME on therapeutic response is also gaining attention in clinical trials evaluating IC blockade treatment in TNBC. Specifically, in the IMpassion130 trial, FI tumors treated with atezolizumab demonstrated significantly extended overall survival (OS) [48].

Delving further, there are prospective avenues yet to be explored, suggesting potential interventions based on the observed TIME heterogeneity across different molecular subtypes of TNBC. In the case of LAR tumors, MDSC-targeted therapies could be of potential value, including inhibition of MDSC function or recruitment into the TIME, inducing their differentiation or mediating their depletion [56,57]. In patients with the MSL subtype, featuring a high number of M2 TAMs, inhibition of the chemokine (C-C motif) ligand 2 (CCL2)/circulating chemokine-receptor-type 2 (CCR2) axis could decrease the recruitment of bone marrow mononuclear cells, subsequently reducing macrophage infiltration in the breast [58,59]. At the same time, activation of the Nuclear factor-κB (NF-κB) pathway could promote the polarization of TAMs to the M1 type, thus impeding the progression of TNBC [60]. Furthermore, by incorporating the aforementioned factors responsible for TIME heterogeneity across TNBC molecular subtypes into clinical practice, we may overcome therapeutic hurdles. A potential resistance to immunotherapy in patients with IM tumors could be addressed by targeting the Wnt/β-catenin pathway [34]. In patients with LAR tumors, targeting metabolic pathways, such as glycolysis or fatty acid metabolism, could also enhance immunotherapy response [61,62,63,64]. In patients with the “immune-cold” M or BL subtypes, targeting immunosuppressive or even immune escape mechanisms could be promising therapeutic options. For instance, potential treatment strategies could include demethylating agents or oncolytic viruses to induce MHC recovery, as well as employing NK cell therapy potentiated by the lack of MHC-I on tumor cells [65,66]. Of note, recent findings reveal that apart from TIME modifications [67,68], TNBC molecular subtypes can change, as well, in a post-treatment manner or over time. For instance, the Lehmann et al. categorization shows that the most common alteration following the neoadjuvant treatment was from BL1 to M subtype [69], with prognostic and therapeutic implications. Thus, in the current era of immunotherapy, integrating insights into molecular pathways offers a strategic approach to optimize therapeutic interventions and improve outcomes for TNBC patients.

### Strengths and Limitations

Strengths of this systematic review include the comprehensive search strategy adhering to PRISMA guidelines, which ensured a rigorous selection process of relevant studies. By focusing on original articles published from 2011, when TNBC molecular subtyping was first introduced [4], we captured the evolving landscape of research in this field over the past decade. Moreover, the inclusion of studies utilizing diverse methodologies for TNBC molecular subtyping and TIME characterization allowed for a more comprehensive analysis of the topic.

The main limitation of the present systematic review is the limited number of studies meeting our inclusion criteria. This may impact the robustness of our findings but also emphasizes the need for further investigation. In addition, due to the limited number of studies included in our analysis, a formal assessment of publication bias was not feasible. Finally, a further limitation is the lack of information on the ethnic origin of the patients in the eligible studies, indicating a critical gap in the existing landscape of TNBC TIME research.

## 5. Conclusions

In conclusion, this systematic review highlights the heterogeneity within TNBC and its complex interplay with TIME. TNBC can be categorized into molecular subtypes that are reproducible albeit different transcriptional methodologies. Each of the TNBC molecular subtypes is characterized by distinct biological features and TIME composition. From “immune-hot” tumors with robust immune infiltration to “immune-cold” tumors with poor immune responses, the variability reflects differential signaling pathway activation, genomic profiles, and metabolic activities across subtypes. Understanding these mechanisms is crucial for tailoring therapeutic strategies, particularly in regard to immunotherapy. Targeting specific pathways responsible for TIME heterogeneity between different TNBC subtypes presents opportunities to optimize treatment and overcome resistance to immunotherapy. Further research into the molecular and immune landscape of TNBC subtypes is warranted to advance personalized therapeutic interventions and improve patient outcomes, possibly taking into consideration the TIME variability of TNBC across diverse populations.

## Figures and Tables

**Table 1:** Summary of studies investigating TIME in relation to TNBC molecular subtypes.

Study (Year)	TNBC Molecular Subtypes	TIME Cellular Evaluation	IC Investigation	Main Results
Bareche et al. 2020 [22]	Lehmann’s (BL1 and BL2, IM, LAR, M, MSL) Jiang’s-FUSCC (BLIS, IM, LAR, MES) Burstein’s (BLIS, BLIA, LAR, MES)	aDCs, iDCs, B cells, NK cells, CD4+ helper (Th1 and Th2), CD8+ T cells, Tcm, Tem, Tfh, γδ T cells, and Tregs, neutrophils, eosinophils, mast cells, macrophages	no	IM subtype: enriched with adaptive immune cells. MSL subtype: enriched with innate immune cells. LAR subtype: enriched with innate immune cells (to a lesser extent than MSL). BL and M subtype: poor adaptive and innate immune responses.
Kim et al. 2020 [23]	MSL, LAR, IM, SL	DCs, B cells, NK cells, CD4+ T cells, Tregs, CD8+ T cells, γδ T cells, plasma cells, neutrophils, eosinophils, mast cells, macrophages	*PD-L1*, *CTLA-4* expression	IM subtype: strongly immune-infiltrated, particularly adaptive immune cells and activated NK cells. MSL subtype: high incidence of M2 macrophages. *PD-L1*, and *CTLA4* significantly enhanced in the IM subtype tumors.
Zhang et al. 2020 [24]	BL1, BL2, IM, M, MSL, and LAR	DCs, Th1 cells, Th2 cells, Tregs, neutrophils, macrophages	*PD-L1*, *PD-1*, *CTLA-4* gene expression	Identification of 8 immune-related hub-genes as prognostic indicators, characterized “immune-hot” status in the TNBC IM subtype. *PD-L1*, *PD-1*, and *CTLA-4* genes more enriched in the IM subtype.
Rodríguez-Bautista et al. 2021 [25]	IM vs. non-IM	CD4+ T cells, CD8+ T cells, Tregs	PD-1, PD-L1, CTLA-4 IHC	IM subtype: enriched with CD8+ TILs and FOXP3+ T-cells. PD-1+ TILs, CTLA-4+ TILs, and PD-L1+ tumor cells increased in the IM subtype.
Thompson et al. 2022 [26]	LAR vs. non-LAR	B cells, NK cells, T cells, CD4+ T cells, CD8+ T cells, plasma cells	no	LAR subtype: lower levels of TILs, increased CD4+ and CD8+ cells, and decreased cycling and regulatory T cells, compared to non-LAR. Non-LAR responders to NAC: increased NK cells, Th cells, cycling T-cells, plasma cells, compared to the non-LAR non-responders.
Suntiparpluacha et al. 2023 [27]	Four subgroups, three of which corresponded to Lehmann’s LAR, BL2, and M subtypes	B cells, CD8+ T cells, neutrophils, MDSCs	*PD-L1*, *PD-1* gene expression	Group 1 (corresponding to LAR subtype): lower amount of CD8+ T cells, MDSCs, B cells, and neutrophils. Group 2 (corresponding to BL2 subtype): enriched with CD8+ T cells and high *PD-L1* and *PD-1* gene expression. Group 3 (corresponding to M subtype): increased neutrophils.

Abbreviations TIME: tumor immune microenvironment; TNBC: triple negative breast cancer; IHC: immunohistochemistry; IC: immune checkpoint; FUSCC: Fudan University Shanghai Cancer Center; BL: basal-like; IM: immunomodulatory; LAR: luminal androgen receptor; M: mesenchymal; MSL: mesenchymal stem-like; BLIS: basal-like immune-suppressed; BLIA: basal-like immune activated; SL: stem-like; aDCs: activated dendritic cells; iDCs: inactivated dendritic cells; NK: natural killer; Th: T helper cells; Tregs: T regulatory cells; Tfh: follicular helper T cells; γδ: gamma delta T cells, PD-1: programmed cell death; PD-L1: programmed cell death ligand; CTLA-4: cytotoxic T-lymphocyte associated protein 4; MDSCs: myeloid-derived suppressor cells; NAC: neoadjuvant chemotherapy.

## Data Availability

No new data were created or analyzed in this systematic review. Data sharing is not applicable to this article.
